# Navigating the Complexities of Managing Severe Anorexia Nervosa in a Medical Ward: A Case Report

**DOI:** 10.7759/cureus.77764

**Published:** 2025-01-21

**Authors:** Khaled Alharmoodi, Mateen Durrani

**Affiliations:** 1 Consultation Liaison Psychiatry, The Lakes Mental Health Hospital, Colchester, GBR

**Keywords:** anorexia nervosa, body mass index (bmi), feeding nasogastric tube, mental health act, multidisciplinary care, refeeding syndrome, severe malnutrition

## Abstract

Anorexia nervosa (AN) is a severe, life-threatening psychiatric disorder often accompanied by significant physical health complications, requiring specialized treatment that is challenging to provide in a general medical setting. This case report presents the complex challenges encountered in managing a 49-year-old woman with severe AN of the purging subtype in a general medical ward setting. Admitted with a life-threatening low body mass index (BMI) of 7.9 and critical physical health issues, her case underscores the intricate relationship between the psychiatric and physical health aspects of AN, especially in an environment with limited specialized resources. The report details her care course, which included medical stabilization, management under the Mental Health Act (MHA), and challenges such as observation, nutritional support, and the ethical complexities surrounding patient autonomy and consent. Additionally, the case highlights systemic limitations, including the lack of specialized training for medical and nursing staff in treating severe AN, as well as the need for multidisciplinary collaboration to navigate risks associated with nasogastric (NG) feeding, refeeding syndrome, and co-occurring psychiatric issues. This report emphasizes the necessity for improved training, specialized resources, and coordinated care in treating complex cases of AN in non-specialized settings to enhance patient outcomes.

## Introduction

Anorexia nervosa (AN) is a severe and complex psychiatric disorder characterized by persistent energy intake restriction, an intense fear of gaining weight, and a distorted body image. It often presents with significant medical complications, including severe malnutrition, electrolyte imbalances, and organ dysfunction. AN is classified into two subtypes: the restricting subtype, where patients limit food intake, and the purging subtype, where individuals engage in compensatory behaviors such as self-induced vomiting or excessive use of laxatives. The purging subtype is particularly challenging, as patients often present with both physical and psychological comorbidities that complicate their management.

This case report explores the significant challenges in treating a 49-year-old woman with a long-standing history of AN, specifically the purging subtype. She was admitted to a general medical ward with life-threatening complications, including a critically low BMI of 7.9 and a weight of 21.8 kg, which highlights the severity of her condition and the urgent need for integrated care. This case exemplifies the intricate relationship between the physical and psychiatric dimensions of AN, particularly in a general hospital setting where specialized eating disorder units are not available. Healthcare teams face multifaceted difficulties in managing the acute medical crises associated with severe malnutrition, while simultaneously addressing the underlying psychiatric disorder. Furthermore, the case underscores the challenges posed by treating such complex patients in non-specialized environments, where medical teams may be inadequately equipped to meet the unique demands of individuals with severe eating disorders [1].

## Case presentation

The patient’s care began with a home visit by the primary care nurse after she reported fatigue and weakness. During this visit, her weight was documented at 21.8 kg, corresponding to a BMI of 9.4 kg/m², a level incompatible with sustaining life. Following consultation with the eating disorder team, it was determined that urgent medical admission was necessary due to her extremely high risk of organ failure.

The patient has struggled to maintain a healthy weight for many years, but her condition worsened considerably after a job loss and hip fracture in March 2024. These events exacerbated her restrictive eating behaviors, leading to severe food restriction, prolonged fasting, self-induced vomiting, and excessive walking to promote weight loss. Before admission, she was persistently walking around her home, expressing a desire to walk long distances despite her deteriorating physical health. While she has a history of laxative abuse, she reported that she was no longer using them.

Upon admission to the general medical unit, she presented with severe physical health complications due to her extreme weight loss. Her electrolyte levels were critically imbalanced, requiring immediate intervention. She had low blood pressure, severe hypoglycemia, and an abnormal ECG indicating a prolonged QTc interval, significantly increasing her risk of sudden cardiac arrest. In the initial days of admission, she refused all interventions except electrolyte correction, declining plans for weight restoration, and medical stabilization. She also refused meal monitoring and the use of a wheelchair, accepting the potential consequences of her condition, stating, "if I die, I die." This response reflected a poor grasp of the urgency of her medical needs and suggested possible impairments in her decision-making capacity regarding her health and safety.

Due to her refusal to engage with the treatment plan and the immediate threat to her life, she was detained under Section 2 of the MHA. Following this, she began eating minimally. While the use of a nasogastric (NG) tube was considered, it was postponed because of her fragile state and her recent decision to eat. The patient appealed her detention, but the Mental Health Tribunal upheld the decision.

During her admission, it was noted that she gained weight rapidly, exceeding 0.5 to 1 kg per week, which raised concerns about refeeding syndrome. However, despite the rapid weight gain and worsening lower limb edema, her echocardiogram (ECHO) remained normal, and her electrolytes were stabilized. The weight gain was attributed to fluid retention. To ensure a more accurate measurement of her BMI, her mid-upper arm circumference (MUAC) was used.

As her admission progressed, the patient developed hospital-acquired pneumonia, which further compromised her health, leading to a peri-arrest event. At this point, palliative care was considered. However, over time, she began to show signs of improvement. She remains hospitalized and continues to receive ongoing treatment.

Psychiatric history

The patient has a long-standing history of AN, specifically the purging subtype, which began at the age of 12. She has undergone multiple admissions to various services, including specialized eating disorder units and psychiatric inpatient care. The majority of these admissions were involuntary under the MHA, with frequent presentations to medical services due to severe weight loss and significant health deterioration. Over the course of her illness, she has developed numerous physical complications, including multiple bone fractures and displacements (involving her hip, wrist, spine, and femur), osteoporosis, chronic hypokalemia, and abnormal ECG readings, notably a prolonged QTc interval, which places her at high risk for sudden cardiac death. She also suffers from chronic fatigue, easy bruising, difficulty with mobility, cold intolerance, hair loss, respiratory issues, reduced height due to osteoporotic fractures, lower limb edema, and persistent low hemoglobin, necessitating intravenous ferritin and blood transfusions.

Her course has been characterized by consistent non-engagement with community mental health and eating disorder services, as well as poor adherence to medical monitoring and treatments. This pattern of disengagement significantly heightens her risk of further health decline and death, as evidenced by her repeated refusal of community-based interventions, failure to attend scheduled appointments, and unresponsiveness to outreach from her care team. Consequently, she has been discharged from several services on multiple occasions due to non-compliance.

## Discussion

Challenges of treating anorexia nervosa in a medical setting

Ethical Considerations in Patient Consent and Autonomy

One of the most significant challenges in managing AN in a medical setting is navigating the complex landscape of patient consent and autonomy. Many individuals with AN may not recognize the severity of their condition, leading to impaired decision-making capacity. Patients often refuse necessary medical interventions, including life-saving treatments, which can be profoundly concerning for healthcare providers. This poses ethical dilemmas about respecting patient autonomy while also ensuring their safety. In some cases, the medical team may be required to intervene under common law, providing life-saving treatment while waiting for an MHA assessment to determine the appropriate legal framework. However, even after a patient is sectioned under the MHA, treatment remains challenging. Forced interventions, though legally permissible, can be countertherapeutic, as they may exacerbate the patient’s distress and reinforce their resistance to care. This creates an ongoing tension between legal mandates and therapeutic goals, making the management of such cases highly complex [2,3].

Placing a Nasogastric Tube

Inserting an NG tube in patients with severe AN as a life-saving intervention presents significant practical challenges. One of the primary concerns is the need to restrain patients who may resist the procedure. In cases of severe AN, physical fragility significantly increases the risks. The process of restraint, particularly in patients with advanced malnutrition, can lead to serious complications, such as bone fractures, dislocations, or soft tissue injuries. These patients often suffer from osteoporosis, making their bones more susceptible to fractures during any physical intervention.

Additionally, the risk of aspiration is heightened in patients with severe AN due to compromised respiratory function from muscle wasting and general physical decline. Improper NG tube insertion or patient resistance during the procedure can result in the tube entering the airway instead of the stomach, increasing the likelihood of aspiration pneumonia. Close monitoring by an experienced medical team is crucial to minimize this risk.

The practical difficulties are compounded by the patient’s resistance and the potential need for repeated attempts, which can further aggravate their physical and psychological condition. As outlined in Nasogastric Tube Feeding Under Restraint: Practical Guidance, when NG tube feeding is performed under restraint, it requires a well-coordinated, multidisciplinary approach to manage both the physical risks and minimize harm. Careful planning and staff training in safe restraint techniques are essential to reduce trauma, but even with these measures, the procedure remains risky and is typically considered only as a last resort [4,5].

Observation in a Medical Setting

Observing patients with eating disorders in a general medical setting poses substantial logistical challenges, particularly in the context of non-compliance and the limitations of non-specialized environments. Unlike psychiatric inpatient units, where continuous monitoring is often facilitated by camera surveillance, medical wards lack such infrastructure, making it difficult to provide constant supervision.

One of the primary challenges is restricting physical activity. Patients with AN often find subtle ways to engage in physical movements, such as walking around, shrugging their shoulders under blankets, or performing exercises while in bed. Without a structured approach to limiting these behaviors, treatment efforts can be significantly undermined. Additionally, the lack of dedicated staff to provide constant observation, especially during critical moments such as meal times or when using the bathroom, where patients may attempt to induce vomiting, further complicates management. The demand for medical staff to care for multiple patients simultaneously exacerbates these challenges, leading to gaps in observation that can jeopardize the effectiveness of treatment and increase the risk of complications [6].

Calculating True Body Mass Index

Accurately assessing a patient’s true BMI in cases of severe AN presents a significant challenge due to the presence of profound physical complications and fluctuating body measurements. Traditional BMI calculations, which rely on height and weight, may fail to accurately reflect the patient’s actual health status, particularly in the context of severe malnutrition and fluid imbalances, such as edema. These imbalances can artificially inflate body weight, leading to misleading BMI results that do not correspond to the patient's true nutritional state.

To address these limitations, alternative methods of assessment, such as using mid-upper arm circumference (MUAC), are often employed to provide a more reliable estimate of the patient’s nutritional condition. This method can offer a more accurate understanding of the severity of malnutrition, guiding more appropriate treatment decisions [7].

Treating Constipation

Addressing constipation in patients with AN, particularly those with a history of laxative abuse, presents considerable challenges. Malnutrition often leads to gastrointestinal dysmotility, making constipation more severe and resistant to conventional treatments. In cases where oral intake is insufficient to stimulate normal bowel function, the management of constipation becomes even more complex. The use of laxatives is particularly problematic, as it can perpetuate maladaptive behaviors and lead to further electrolyte imbalances, dehydration, or dependence on purging mechanisms. Healthcare providers must carefully balance the need for effective constipation relief while minimizing the risk of reinforcing harmful behaviors. In instances where laxatives are indicated, they should be administered under supervision to ensure that misuse does not occur [8].

Lack of Experience Among Medical Staff

An additional challenge in treating patients with eating disorders in a medical setting is the limited specialized expertise among both medical doctors and nursing staff. Many healthcare professionals, including doctors, may not have received sufficient training in managing AN, which can lead to mismanagement of both the physical and psychiatric aspects of care. This lack of experience may result in misconceptions about the disorder, inappropriate interventions, or inadequate support for the patient’s complex needs.

For nursing staff, these limitations present additional challenges, particularly when it comes to the practical aspects of care, such as monitoring food intake, providing close observation, and ensuring compliance with treatment protocols. Patients with AN may exhibit non-cooperative behaviors, including resistance to eating plans, attempts to evade supervision, or refusals of necessary medical interventions, adding to the complexity of nursing care. These factors can lead to increased frustration and difficulty in maintaining consistent care, especially within the time and resource constraints of a general medical setting.

To address these challenges, it is essential for both medical and nursing teams to collaborate closely with eating disorder specialists, incorporate updated knowledge on evidence-based practices and receive ongoing training tailored to the unique demands of treating severe AN. These efforts are critical to enhancing team preparedness and ultimately improving patient outcomes in complex cases [9,10].

Addressing Co-occurring Mental Health Issues

Many patients with AN present with co-occurring mental health conditions, such as anxiety, depression, or obsessive-compulsive disorder (OCD). These psychiatric comorbidities can complicate the management of AN, as they may intensify the severity of the eating disorder or impede the patient’s ability to engage in the therapeutic process. For example, a patient with OCD may experience intrusive thoughts about food and weight, further entrenching maladaptive behaviors and obstructing recovery efforts. However, treating co-occurring psychiatric conditions in patients with severe AN within a medical setting presents additional challenges. Many psychotropic medications commonly used to manage anxiety, depression, or OCD may have adverse effects on physical health, particularly when the patient is physically compromised. For instance, certain medications may exacerbate electrolyte imbalances or cardiovascular risks, further complicating the patient’s medical management. Balancing the treatment of psychiatric comorbidities with immediate physical health concerns necessitates close collaboration between medical and psychiatric teams to ensure that interventions do not compromise the patient’s safety while addressing both their physical and mental health needs [11,12].

Environmental Factors and Medical Settings

The typical medical environment is often ill-suited for the effective treatment of patients with AN. These individuals require a therapeutic setting that fosters a sense of safety, support, and a positive relationship with food elements that are critical to their recovery. However, the sterile and clinical atmosphere of a general medical ward may heighten feelings of anxiety and distress, which can hinder the patient’s ability to engage with treatment. Creating a supportive environment that accommodates the unique psychological and nutritional needs of patients with AN is essential but remains challenging within the limitations of a standard medical setting. This lack of specialized resources and focus on emotional well-being may inadvertently undermine the recovery process.

Multidisciplinary Team Collaboration

Effective treatment of AN in a medical setting requires a collaborative, multidisciplinary approach. This team typically includes medical doctors, psychiatrists, nurses, dietitians, eating disorder specialists, and occupational therapists, each contributing essential expertise to address the complex nature of AN. However, coordinating the efforts of such diverse professionals poses significant challenges, especially as most eating disorder specialists do not operate within general hospitals. This disconnect can lead to gaps in care and delays in implementing appropriate interventions. The situation is further complicated by the frequent rotation of medical teams in hospital settings, making it even more challenging to maintain continuity of care. Regular multidisciplinary meetings are not always feasible, which exacerbates the difficulties in providing consistent, patient-centered treatment in such environments [13].

## Conclusions

The management of severe AN in a medical ward presents significant challenges, as illustrated in this case. Treating life-threatening physical complications while addressing complex psychiatric needs requires specialized expertise often lacking in general hospital settings. Multidisciplinary collaboration is crucial but can be difficult due to communication barriers, frequent team changes, and the absence of specialized eating disorder services. This case emphasizes the need for better training of medical staff, improved access to eating disorder specialists, and environments tailored to the unique needs of AN patients. A coordinated, patient-centered approach is essential to improving outcomes in such complex cases.
